# Phenotypical differences of neutrophils patrolling tumour-draining lymph nodes in head and neck cancer

**DOI:** 10.1038/s41416-024-02891-5

**Published:** 2024-11-14

**Authors:** Sandra Ekstedt, Krzysztof Piersiala, Aeneas Kolev, Pedro Farrajota Neves da Silva, Gregori Margolin, Susanna Kumlien Georén, Lars-Olaf Cardell

**Affiliations:** 1 Division of ENT Diseases, Department of Clinical Sciences, Intervention and Technology, Karolinska Institutet, Stockholm, Sweden; 2 Department of Otorhinolaryngology, Karolinska University Hospital, Stockholm, Sweden; 3 Medical unit Head Neck, Lung and Skin Cancer, Karolinska University Hospital, Stockholm, Sweden; 4 Department of Pathology and Cytology, Karolinska University Hospital, Stockholm, Sweden

**Keywords:** Oral cancer, Granulocytes, Lymph node

## Abstract

**Background:**

The complexity and heterogeneity of neutrophils are recognized, especially their roles in modulating inflammation and cancer immune responses. The detailed functions of neutrophils in human tumour-draining lymph nodes (TDLNs), specifically in the context of head and neck cancer, remain inadequately characterized.

**Aim:**

This study aims to delineate the phenotypic diversity of neutrophils in TDLNs, non-tumour-draining lymph nodes (nTDLNs) from patients with oral squamous cell carcinoma (OSCC), and to evaluate their correlation with clinical outcomes.

**Methods:**

A flow cytometry-based investigation.

**Results:**

Neutrophils manifest a tissue-specific heterogeneity with significant phenotypic differences between compartments. A substantial fraction of neutrophils displayed an activated CD16^high^CD62L^dim^ profile in TDLNs, more prominent in patients with advanced T stages, implicating their involvement in the disease’s progression. Notably, the presence of this activated neutrophil phenotype in TDLNs was strongly associated with poorer patient prognosis.

**Conclusions:**

The study confirms the heterogeneity of neutrophils in human TDLNs, aligning with findings from animal models but extending them to show clinical relevance in human disease. The correlation of neutrophil phenotypes with cancer progression and prognosis emphasizes the importance of these cells in the tumour-microenvironment. The data suggests a future possibility to develop targeted therapies that modulate the neutrophilic response in OSCC.

## Introduction

Neutrophils are the most abundant immune cell type in peripheral circulation. They are rapidly recruited into sites of inflammation and show strong effector responses. Neutrophils have for a long time been considered a homogenous, short-lived and non-complex cell type. However, over the years there has been increasing evidence that they exhibit numerous phenotypes, shape inflammation, cancer immune responses and can interact with other immune cells belonging to the adaptive immune system [1–5]

How exactly neutrophils shape adaptive immune responses in humans remains still understudied. In one of our previous studies, we showed that activated neutrophils exhibit a T-cell priming capacity and an ability to enhance eosinophil migration in vitro [6]. Others also proved that neutrophils interact with antigen-presenting cells and T-lymphocytes [7, 8]. Interestingly, neutrophils were shown to increase in numbers in secondary lymphatic organs during inflammation and cancer [9, 10]. Furthermore, neutrophils are the first cell type to arrive in tissue-draining lymph nodes after antigenic challenge [11, 12]. Neutrophils have been observed to carry antigens [12], express molecules essential for antigen presentation and co-stimulation [12], and release chemokines that influence adaptive immune cells. The presence of neutrophils in lymph nodes suggests that they may play a role of immune response regulators rather than patrolling for pathogens in secondary lymphoid organs. However, this hypothesis awaits validation through further investigation in human samples.

Tumour-draining lymph nodes (TDLNs) are crucial organs in orchestrating anti-cancer immunity [13]. It is known that neutrophils accumulate in TDLNs in different cancer types, including head and neck cancer [14]. The presence of neutrophils in TDLNs has been associated with a worse prognosis [15]. It has been also shown that neutrophil extracellular traps (NETs) facilitate metastases seeding and migration of cancer cells via the endothelial barrier in the lung cancer model [16]. Recently, Pyaleva et al. showed that TDLNs-associated neutrophils prime anti-cancer T cell responses in early stages of head and neck cancer, but in more advanced stages they induce an immunosuppressive environment within TDLNs [17].

Despite accumulating evidence supporting the importance of neutrophils in the development of anti-cancer immunity, the exact role of neutrophils in TDLNs, particularly in humans, remains vastly unexplored. The majority of novel concepts in neutrophil biology, especially related to their interactions with the lymphatic system, have been discovered in mouse and animal models. This is why, we here present a study investigating neutrophils in the subcapsular area of human tumour-draining lymph nodes of patients with oral squamous cell carcinoma (OSCC). The project aims to investigate whether there are any phenotypical differences based on the expression of CD16, CD62L, CD184, CD11b, CD36, and CD47 between tumour-associated neutrophils (TANs), neutrophils in TDLNs, non-TDLNs in patients with OSCC as well as in lymph nodes coming from the neck of patients without cancer.

## Materials and methods

### Patient characteristics

Eligible patients enroled for this study met following inclusion criteria: (1) diagnosis of primary or recurrent oral cancer squamous cell carcinoma (OSCC), (2) tumour/recurrence excision with sentinel node assisted elective neck dissection (identification in SPECT-CT, and location confirmed intraoperatively by gamma probe) performed at Karolinska University Hospital, Stockholm, Sweden between March 2019 and June 2022 3) willingness to participate in the study. For details regarding setting of sentinel node procedure at Karolinska University Hospital, please see paper of Kågedal et al. [18]. Exclusion criteria were as follows: (1) systematic autoimmune diseases (2) synchronous second malignancies, hemo-lymphopoietic malignancies in the past (3) any other acute or chronic condition that could influence immunological milieu in lymph nodes. In addition, we analyzed 12 neck lymph nodes from patients with benign salivary gland tumours. At our centre, the sentinel node protocol is the standard approach. For patients included in this study, 1–2 additional lymph nodes that tested negative during the sentinel node detection procedure were harvested from the same incision used for sentinel node retrieval and were classified as non-tumour draining lymph nodes (nTDLNs). Within the 12 patients who contributed control lymph nodes from neck, the underlying pathologies were diverse: four patients had pleomorphic adenoma of the parotid, three had lateral cervical cysts, two had sialadenitis, one had a lipoma in the neck, and one had hyperplastic oncocytosis of the parotid gland. These lymph nodes are further called as “healthy lymph nodes” (hLNs). Patients who contributed with hLNs did not meet any exclusion criteria.

Lymph nodes from patients with benign conditions can be considered appropriate controls in this setting for several reasons. First, the absence of malignant disease in these nodes suggests that the immune response within the lymph nodes is not skewed by oncogenic processes, thus reflecting a more “normal” or non-tumour-influenced immune environment. Despite the presence of benign pathology in adjacent tissues (such as pleomorphic adenoma or sialadenitis), these conditions do not typically induce the profound immune dysregulation seen in malignant diseases. Therefore, the lymph nodes from these patients can still provide valuable insight into baseline neutrophil characteristics and immune function. However, it is essential to acknowledge the potential limitations. Even benign conditions can result in localized inflammation or mild immune activation, which might slightly alter neutrophil behaviour or other immune parameters within the lymph nodes.

### Tissue preparation for neutrophil analyses

**Fig. 1:**
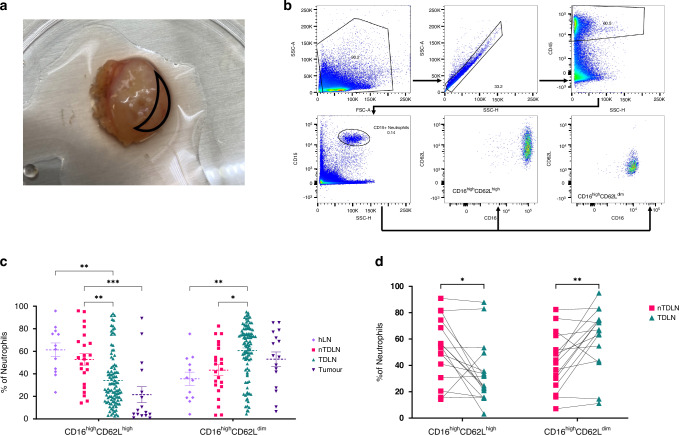
Analysis of CD16 and CD62L Expression on Neutrophils in Lymph Node. **a** lymph node specimen used within the project. Part taken for downstream analysis is marked with black lines. **b** Simplified gating strategy for detection of CD16^high^CD62L^high^/CD16^high^CD62L^dim^ populations. **c** Comparison of CD16^high^CD62L^high^ and CD16^high^CD62L^dim^ populations between hLNs (*n* = 12), n-TDLNs (*n* = 23), TDLNs (*n* = 102) and tumour samples (*n* = 16). (**d**) Paired analysis comparing the percentage of CD16^high^CD62L^high^/CD16^high^CD62L^dim^ populations between TDLNs and n-TDLNs coming from the same patient (*n* = 16) and same region of the neck.

The unfixed neck sample after excision were transferred directly to Pathology Department, where one of the designated pathologists handled samples and separated lymph nodes parts and a sample of the tumour (all TDLNs and 1-2 nTDLNs per one patient). The lymph nodes after surgical excision were kept in pre-chilled MACS Tissue Storage Solution and used within 1 hour for further analysis. In this project, we used sections of the lymph node containing the subcapsular sinus and parts of the interfollicular zone for downstream analysis, as illustrated in Fig. 1a. The tissues were then put through a 100 µm cell strainer (BD biosciences #352360) and washed with RPMI 1640 (Invivogen, San Diego, CA, USA). This protocol exclusively utilized mechanical dissociation to achieve a single-cell suspension. Within the study, 23 nTDLNs, 102 TDLNs, 12 hLNs and 16 tumour samples were analysed.

### Flow cytometry

Single-cell suspensions were resuspended in PBS and incubated with FC-block for 5 minutes. The cells were then incubated with antibodies seen in supplementary Table 1 at room temperature in the dark for 20 minutes and washed with PBS. Cells were resuspended in PBS with 1% paraformaldehyde (HistoLab #02178). All antibodies used, have been titrated for optimal concentration. The fixed sample were analyzed on LSR FORTESSA x20 (BD Biosciences). Analysis of the flow cytometry data was performed with FlowJo version 10.7.1 (LLC, USA). The gating strategy for the identification of neutrophils is summarized in Fig. 1.

### UMAP and PhenoGraph

First, three random samples from TDLN, n-TDLN, and hLN were imported into the Flowjo software for phenotype characterization. Neutrophils were gated as previously described. Next, the gated neutrophils were exported to generate a new file. Then, the samples in the new file were individually normalized to 1780 cells in nTDLNs, 3000 cells in hLNs, and 3000 cells in TDLNs using the downsample plugin (v.3.3.1). Thus, the nTDLNs were underrepresented in the downstream analysis. Still, the number of events was sufficient to create a reliable result in clustering. The samples were subsequently exported and put together using the concatenation tool in FlowJo. A UMAP was performed to reduce the dimensions of the multiparameter data (FlowJo v.10.8.1). Clusters of phenotypically related cells were then detected using FlowJo plugin PhenoGraph (v.2.5.0) [19]. PhenoGraph was run with the following parameters: CD16, CD62L, CD11b, CD184, CD36 and CD47; K = 107.

### Statistical analysis

Statistical analyses were performed with GraphPad Prism version 9.0.0 (GraphPad Software, La Jolla, CA, USA). The Kolmogorov-Smirnov normality test was used to determine if data sets were normally distributed, and Mann-Whitney or two-tailed Unpaired t-test were chosen, depending on the distribution of the data. Paired t-test was used to compare paired groups of data. *P* < 0.05 (*) was considered significant, and *P* < 0.01 (**), *P* < 0.001 (***), *P* < 0.0001 (****) were considered highly significant.

## Results

### Patient clinical and pathological characteristics

The cohort consisted of 37 individuals with OSCC, with a slightly higher representation of males (54.1%) compared to females (45.9%). Smoking history revealed that 56.8% were previous or current smokers, while 43.2% had never smoked. Tumour sites varied, with the majority located in the mobile tongue (62.16%), followed by gingiva (21.62%), floor of the mouth (8.11%), and buccal mucosa (8.11%). The pathological T status distribution was as follows: 13 patients with pT1 (35.14%), 11 with pT2 (29.73%), 7 with pT3 (18.92%), and 6 with pT4 (16.21%). Ten patients (27%) had nodal metastases confirmed in pathology. Regarding recurrence, 70.3% of patients were recurrence-free, while 29.3% experienced recurrence during the study period. Please see the summary of data in Table 1 and the complete data on all subjects in Supplementary Table 2.

**Table 1:** Demographic characteristics and clinicopathological data of enroled patients.

Variable	*N* (%)
**Age (mean** **±** **SD)**	68.5 ± 10.8
**Sex**	
Female	17 (45.9)
Male	20 (54.1)
**Smoking history**	
Never smoker	16 (43.2)
Previous/current smoker	21 (56.8)
**pT status**	
pT1	13 (35.14)
pT2	11 (29.73)
pT3	7 (18.92)
pT4	6 (16.21)
**pN status**	
N0	27 (73.0)
N+	10 (27.0)
**Tumour site**	
Mobile tongue	23 (62.16)
Gingiva	8 (21.62)
Floor of the mouth	3 (8.11)
Buccal mucosa	3 (8.11)
**Recurrence status**	
Recurrence-free	26 (70.3)
Recurrence	11 (29.3)

### Neutrophils in TDLNs exhibit tumour-alike subsets in contrary to non-TDLNs and healthy lymph nodes

Within the study, 23 nTDLNs, 102 TDLNs, 12 hLNs and 16 tumour samples were analysed. Neutrophils were more abundant in TDLNs (on average 2558 ± 5460 neutrophils per sample) as compared to nTDLNs (on average 1774 ± 1783 neutrophils per sample). However, no normalization was applied to the samples, and the size and weight of lymph nodes varied significantly between patients. Therefore, this observation should be interpreted with caution. First, the activation phenotypes based on expression of CD16 and CD62L were investigated. Neutrophils expressing high levels of CD16 and CD62L (CD16^high^CD62L^high^) are mature and nonactivated, whereas the CD16^high^CD62L^dim^-expressing neutrophils are believed to be mature and activated [1]. The majority of neutrophils found in hLNs (61.25% ± 21.13) and nTDLNs (52.82% ± 24.24) were CD16^high^CD62L^high^. In contrary to TDLNs and tumour samples, where the majority of neutrophils were CD16^high^CD62L^dim^ (60.58% ± 23.84 and 53.02% ± 25.52, respectively). nTDLNs were characterized by a significantly higher level of CD16^high^CD62L^high^ neutrophils compared to TDLNs and tumour samples (p values: 0.0050 and 0.0005, respectively). On the other hand, TDLNs were characterized by significantly higher levels of CD16^high^CD62L^dim^ neutrophils as compared to nTDLNs and hLNs (p values: 0.0115 and 0.0044, respectively)(Fig. 1c). Furthermore, within the study material, we gathered 16 pairs where we had TDLN and nTDLN coming from the same patient and from the same region of the neck, meaning the distance from the tumour to the lymph node was approximately the same. The levels of CD16^high^CD62L^high^ neutrophils were significantly lower in TDLNs as compared to their paired nTDLNs (p = 0,0106) and levels of the CD16^high^CD62L^dim^ were significantly higher in TDLNs compared to nTDLNs (*p* = 0,0092) (Fig. 1d).

### Neutrophil exhibit more activated phenotypes in TDLNs with increasing T stage

**Fig. 2:**
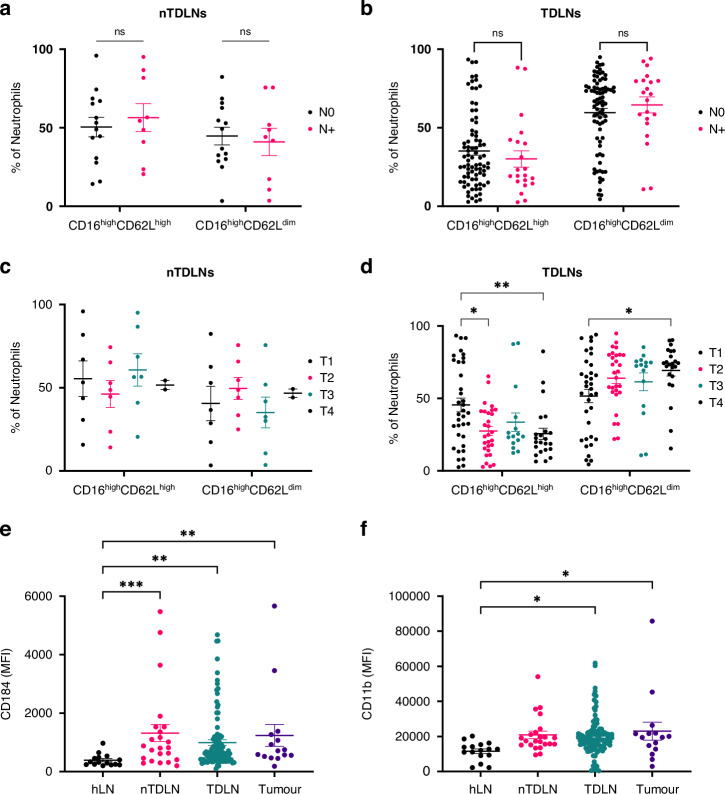
Analysis of CD16 and CD62L Neutrophil Subopulations and Marker Expression in Lymph Nodes. Comparison of CD16^high^CD62L^high^/CD16^high^CD62L^dim^ populations between patients with N0 and N+ stage in nTDLNs (**a**) and TDLNs (**b**). Comparison of CD16^high^CD62L^high^/CD16^high^CD62L^dim^ populations between patients with T1-T2 stage and T3-T4 stage in nTDLNs (**c**) and TDLNs (**d**). **e** Expression of CD184 across analyzed compartments. The number of samples was as follows: hLNs (*n* = 12), n-TDLNs (*n* = 23), TDLNs (*n* = 102) and tumour samples (*n* = 16). **f** Expression of CD11b across analyzed compartments. The number of samples was as follows: hLNs (*n* = 12), n-TDLNs (*n* = 23), TDLNs (*n* = 102) and tumour samples (*n* = 16)

The association between activation phenotypes and TNM staging was investigated. There were no differences in levels of CD16^high^CD62L^high^ and CD16^high^CD62L^dim^ in nTDLNs and TDLNs between patients with and without nodal involvement (pN0 vs pN + ) (Fig. 2a, b). However, in TDLNs the levels of CD16^high^CD62L^high^ were significantly lower in higher T stages and the proportion of CD16^high^CD62L^dim^ neutrophils increased in higher T stages. This observation was not present in nTDLNs (Fig. 2c, d).

### Neutrophils in patients with cancer express higher levels of CD184 (CXCR4) and CD11b

Neutrophils expressing CD184 were shown previously to be effective in migration to the inflammatory sites and to have high phagocytic activity [20]. Neutrophils in nTDLNs, TDLNs and in the tumour expressed more abundantly CD184 compared to healthy lymph nodes from patients without a malignancy. The same trend was observed for the expression of activation marker CD11b, where significantly higher levels of expression were observed in TDLNs and tumour samples when compared to hLNs.

### Identification of unique subsets of neutrophils in healthy lymph nodes, nTDLNs and TDLNs

**Fig. 3:**
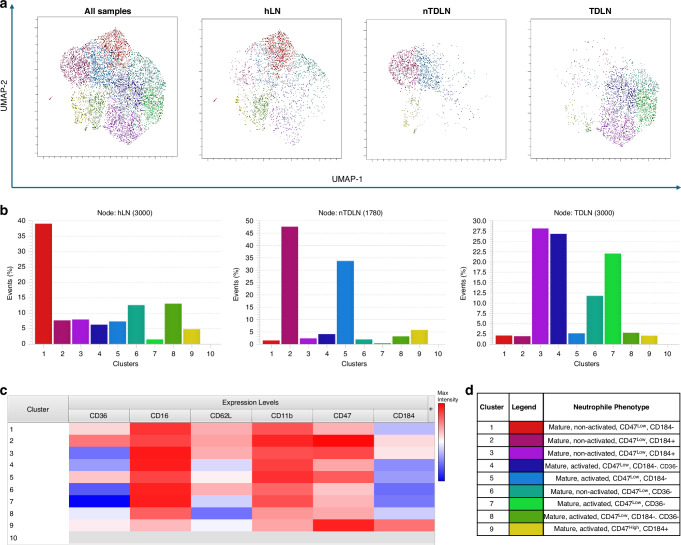
Characterization of Neutrophil Clusters in Lymph Node Compartments. **a** UMAP plot of various neutrophil cell clusters obtained from three pooled samples of hLN, nTDLNs and TDLNs. Each colour represents a different cell cluster (identified by PhenoGraph) and each dot represents a single cell. **b** The bar chart shows the frequency (%) of each cluster in the investigated samples. **c** Heatmap of median marker expression across all clusters. **d** Legend summarizing the phenotypic characteristics of the clusters.

To delineate the distinctions in neutrophils phenotypes between hLNs, nTDLNs and TDLNs, we used a dimensional reduction analysis called a uniform manifold approximation and projection (UMAP). Further, we used the clustering algorithm PhenoGraph to cluster cells based on expression of six surface markers of interest. These markers on neutrophils were as follows: CD16, CD62L, CD11b, CD184, CD36 and CD47. The PhenoGraph identified 9 different clusters based on similarity of marker expression patterns. There were striking differences in neutrophils phenotypes between hLNs, nTDLNs and TDLNs (Fig. 3a). The bar chart shows the frequency (%) of each cluster in the investigated samples (Fig. 3b). Using ClusterExplorer, the heatmap showing the relative intensity of each parameter for a given cluster was calculated and is presented in Fig. 3c. In this dimensional reduction analysis, we added two markers on neutrophils, namely CD36 and CD47. High expression of CD47 on neutrophils is believed to be sign of a prolonged survival of neutrophils in tissues [21]. CD36 is a scavenger receptor, which regulates uptake of fatty acid and balances the intracellular lipid content. Al-Khami et al. showed that deletion of CD36 could delay tumour growth through a CD8+ T cell-mediated response [22].

Neutrophils within cluster 1 can be defined as normal, non-activated neutrophils as they were of CD16^high^CD62L^high^ phenotype with positivity for CD36 and relatively low expression of “don’t eat me” CD47. In hLNs, cluster 1 was the most abundant. Within nTDLNs, clusters 2 and 5 were the most abundant. Cluster 2 represents as mature, nonactivated neutrophils (CD16^high^CD62L^high^) but in contrary to cluster 1, cells were characterized by positivity for CD47 and CD184. This could represent a migratory, patrolling subset of neutrophils. Cluster 5 represents mature, activated neutrophils (CD16^high^CD62L^dim^), negative for CD184 and positive for both CD36 and CD47. This could represent stationary neutrophils in the lymph node, ready for phagocytosis. TDLNs were characterized by abundance of different clusters. Clusters 3, 4, 7 and 6 were the most abundant ones. The common feature for these clusters was negativity for CD36. Furthermore, clusters 3,4, and 7 represented mature activated neutrophils (CD16^high^CD62L^dim^). Clusters 4,6 and 7 were relatively low in expressing CD184. The most abundant cluster 3 was strongly positive for CD47. Taken together, these clusters may represent activated neutrophils, which are meant to stay for a longer period in TDLNs, as they express “don’t eat me signals” and some clusters are low in expression of the migratory molecule CD184.

### Patients with higher levels of CD16^high^CD62L^dim^ neutrophils in TDLNs, higher T stage and nodal involvement have worse prognosis

**Fig. 4:**
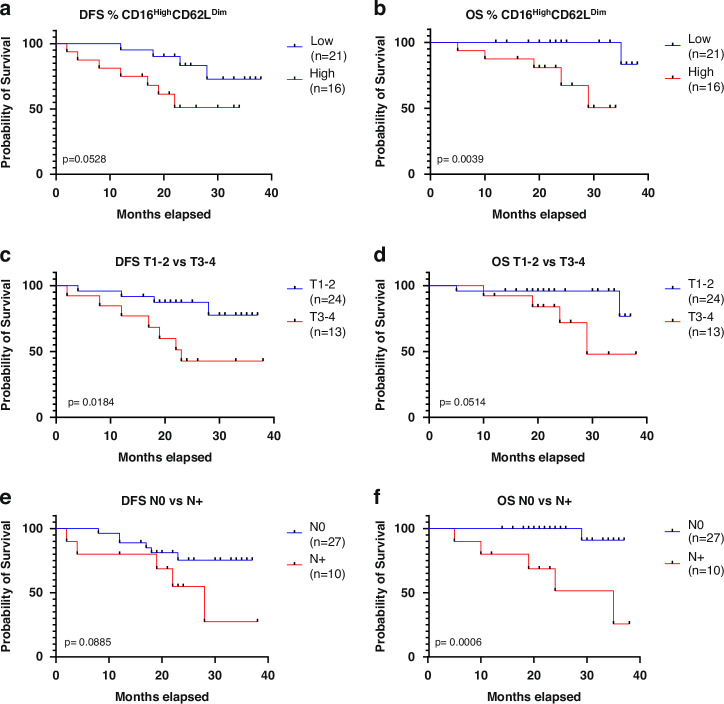
Kaplan-Meier Survival Curves for DFS and OS in Relation to CD16highCD62Ldim Neutrophil Populations in Lymph Nodes and Tumor Staging. Kaplan-Meier curves showing DFS and OS in relation to the level of CD16^high^CD62L^dim^ population in TDLNs (**a**, **b**), T stage (**c**, **d**) and N stage (**e**, **f**).

To conduct survival analysis, we categorized the level of CD16^high^CD62L^dim^ expression into low and high groups, using the median expression in all TDLNs as the threshold. In patients who contributed with more than one TDLN, a mean level of CD16^high^CD62L^dim^ was calculated. Using Kaplan-Meier method, the disease-free survival (DFS) and overall survival (OS) were calculated for CD16^high^CD62L^dim^ Low/High group, T1-T2 vs T3-T4 tumours and patients with N0 vs N+ stage. As shown in Fig. 4, patients with larger tumours had significantly worse DFS compared to patients with T1-T2 stages (log-rank test, *p* = 0.0184). Patients with High levels of CD16^high^CD62L^dim^ and N+ status showed a trend to worse DFS, however these comparisons did not reach a statistical significance (*p* values: 0.0528 and 0.0885, respectively) (Fig. 4 a, c, e). For OS, patients with high levels of CD16^high^CD62L^dim^ in TDLNs and N+ stage showed significantly worse OS (log-rank test, *p* values: 0.0039 and 0.0006, respectively)(Fig. 4b, f). There was a trend for worse OS in patients with larger tumours (T3-T4), however it did not reach a statistical significance (*p* = 0.0514)(Fig. 4d). To further extend the findings, the multivariate Cox proportional-hazards models for DFS and OS were calculated. None of the studied variables showed to be an independent prognostic factor for DFS or OS.

## Discussion

Our findings provide evidence of neutrophil residency within the subcapsular regions of both TDLNs and nTDLNs in patients suffering from OSCC. These immune cells were also detected in corresponding regions in hLNs extracted from individuals undergoing surgical intervention for benign salivary gland lesions. We also demonstrate a distinct variance in maturation and activation states of neutrophils, as delineated by the expression of CD16 and CD62L markers, across these anatomical sites. In hLNs and nTDLNs, neutrophils predominantly exhibited a mature, non-activated phenotype, contrasting with the mature, activated phenotypes prevalent in TDLNs and tumour samples. This suggests that neutrophils within TDLNs more closely mirror the characteristics of TANs rather than those found in hLNs. Further, the elevated expression of CD47 in TDLNs and lower level of CD36 on TDLN-resident neutrophils, indicating a potential mechanism for their extended longevity within the TDLN microenvironment, a notable deviation from the typical transient nature of these cells. The extended lifespan is especially relevant given that the elevated presence of CD16^high^CD62L^dim^ neutrophils in TDLNs is associated with a less favourable patient prognosis.

Previously, there was a longstanding belief that neutrophils were excluded from lymphatic vessels and secondary lymphoid organs. This view was recently challenged by multiple studies confirming the presence of neutrophils in lymph nodes and lymphatic fluid, both in homoeostasis and during disease [18, 23–25]. What is more, neutrophils were showed to be the first cell type arriving at the draining lymph nodes in response to *S. aureus* challenge and to have the capacity to transfer particles of the bacteria to the node [12]. The neutrophils within lymph nodes were predominantly observed in the subcapsular sinus as this is the entrance point for cells arriving via afferent lymphatic vessels [11, 24]. Some neutrophils were also observed in the interfollicular zone located directly under the subcapsular sinus in close proximity to B- and T cells [26]. T cell zone of the lymph node was shown to be poor in neutrophils. This is why, within the scope of this project, we decided to investigate parts of the lymph node containing subcapsular sinus and parts or whole of the interfollicular zone. Please see Fig. 1a for details. Our study confirms that neutrophils are present and detectable by flow cytometry in healthy human lymph nodes in homoeostasis and in lymph nodes coming from patients suffering from cancer. In our cohort, as anticipated, neutrophils in TDLNs were more abundant than in nTDLNs. This observation may have a potential clinical significance, as previously, higher numbers of neutrophils in TDLNs in patients suffering from gastric cancer and OSCC, were associated with more advanced stages of the disease and poorer prognosis [14, 27].

Beyond confirming the presence of neutrophils in TDLNs, our study adds knowledge about striking phenotypic differences of neutrophils between different anatomical locations, namely different types of lymph nodes and tumour samples. Using well described neutrophils markers CD16 and CD62L, we applied a classification introduced by Pillay et al. [1], where CD16^high^CD62L^high^ neutrophils are considered to be mature, non-activated subset, and CD16^high^CD62L^dim^ are defined as mature, activated neutrophils. Elevated levels of CD16^high^CD62L^dim^ neutrophils were found previously in blood of patients with systemic inflammation and in malignancies [28]. Furthermore, Pillay et al. showed that CD16^high^CD62L^dim^ neutrophils exhibit immunosuppressive activity on T cells [29]. Our group [4], reported previously that higher levels of CD16^high^CD62L^dim^ neutrophils in the blood of patients suffering from HNSCC correlated with better prognosis, whereas others showed [30] correlation with unfavourable prognosis in patients with chronic lymphocytic leukaemia (CLL). Nevertheless, phenotypic differences based on these markers were not previously studied in human TDLNs. We found that a high fraction of neutrophils found in TDLNs exhibited CD16^high^CD62L^dim^ phenotype. Surprisingly, the level of CD16^high^CD62L^dim^ neutrophils was higher in TDLNs as compared to tumour samples. Healthy lymph nodes and nTDLNs were characterized by significantly lower levels of CD16^high^CD62L^dim^ neutrophils and the majority of found neutrophils in these locations were mature nonactivated (CD16^high^CD62L^high^). Within this project, we investigated the frequency of CD16^high^CD62L^dim^ neutrophils in lymph nodes coming from the same patient and the same anatomical location (region of the neck), with the only difference that one of the lymph nodes was a TDLN having direct contact with a tumour (confirmed in the sentinel node biopsy method) and the other one did not have direct contact with the tumour. What we observed was that draining the tumour directly was associated with a significantly higher level of CD16^high^CD62L^dim^ neutrophils. This observation suggests that the composition and activation of neutrophils in the TDLNs is influenced by the crosstalk with the primary tumour.

Furthermore, we found that level of CD16^high^CD62L^dim^ neutrophils in TDLNs correlated with the size of the tumour (T stage) but not with a N stage. Interestingly, such an observation was not present in nTDLNs. This could suggest that phenotypes of the neutrophils in TDLNs correlate with the advancement of the disease. This was further confirmed in survival analysis showing that high levels of CD16^high^CD62L^dim^ neutrophils in TDLNs correlated with worse prognosis, but this finding turned out not be independent in multivariate analysis when T stage and N stage were taken into account as confounding factors.

To further investigate differences between neutrophils in lymph nodes, we applied a dimensional reduction analysis and cell clustering software to find distinct phenotypic differences. As shown in Fig. 3, neutrophils in hLNs, nTDLNs and TDLNs showed completely different profiles of expression of studied markers (CD16, CD62L, CD11b, CD184, CD36 and CD47). Thus, our findings confirm tissue-specific plasticity and neutrophil heterogeneity within human lymph nodes. Neutrophils present in TDLNs had higher expression of CD47 molecules, which can indicate their potentially prolonged survival in TDLNs. This can have striking consequences when discussing the role of neutrophils as immune system regulators. Animal studies showed that neutrophils interact with T cells within lymph nodes, but the majority of interactions are transient, usually lasting less than 1 minute [31]. If the neutrophils stay longer in TDLNs, there is a chance for prolonged interaction between neutrophils and other cells present in the lymph nodes.

Knowing that neutrophils are present in TDLNs, it is warranted to further investigate these cells in human samples. Despite accumulating number of reports, the function and role of neutrophils in TDLNs is still unclear. Numerous studies showed in vitro that neutrophils have the capacity to shape both innate and adaptive responses, however it is still unknown whether this process take places in TDLNs. Nevertheless, the proximity of neutrophils to macrophages, T- and B cells within lymph nodes suggests that it is possible that neutrophils serve as immune system regulators and further investigation is needed in order to examine therapeutic potential of targeting lymph nodes associated neutrophils.

The finding that mature, activated neutrophils are more prevalent in tumour-draining lymph nodes (TDLNs) compared to controls may have significant translational implications. Activated neutrophils are key players in immune regulation and can contribute to an immunosuppressive microenvironment that favours tumour growth and immune evasion. Their increased presence in TDLNs suggests a potential mechanism by which tumours modulate local immune responses to escape immune surveillance. This insight opens up possibilities for targeting neutrophil activation as part of therapeutic strategies, particularly in high-risk OSCC patients. By inhibiting neutrophil-driven immunosuppression, we may be able to improve the effectiveness of immune-based therapies and enhance patient outcomes in OSCC.

While our study provides valuable insights into the biology of neutrophils in TDLNs, it is important to acknowledge certain limitations that may impact the interpretation of our findings. First, the sample size for our investigation was relatively modest, consisting of 37 participants. However, we believe that the number of analyzed samples, including 102 TDLNs, adequately represents the basis for our drawn conclusions. Furthermore, within this project we performed only flow cytometric analysis of dissociated samples. Thus, the spatial aspect was impossible to be further investigated in the same patients. As a result, we are not able to describe the exact location within the lymph node of the studied cells and their proximity or distance to other cell types. Addressing these challenges offers opportunities for refinement in future investigations.

## Conclusions

In summary, the presented data shows the presence of neutrophils in the subcapsular region of human lymph nodes. These neutrophils manifest a tissue-specific heterogeneity, with those in TDLNs demonstrating an activated phenotype. Notably, this activation may be associated with an extended lifespan compared to their counterparts in hLNs or nTDLNs. The activated neutrophils, characterized by CD16^high^CD62L^dim^ expression, correlated significantly with tumour stage, suggesting an involvement in the pathogenesis and progression of cancer. This association with the tumour stage and prognosis not only implicates that neutrophils are important players in the tumour microenvironment but also, they can be considered as potential prognostic biomarkers in OSCC.

## Supplementary information

Supplementary tables

## Data Availability

All data generated or analyzed during this study are included in this published article and its supplementary information files.
