# Supplementary material for: Phenotypical differences of neutrophils patrolling tumour-draining lymph nodes in head and neck cancer
Source: Br J Cancer. 2024 Nov 14;131(12):1893–900. doi: 10.1038/s41416-024-02891-5 (PMC11628601; doi:10.1038/s41416-024-02891-5)
Supplement: Supplementary file 1 — Supplementary tables [file 41416_2024_2891_MOESM1_ESM.docx]

| ANTIGEN | SUPPLIER/REFERENCE | CLONE | FLUOROCHROME |
| --- | --- | --- | --- |
| CD15 | BD/563142 | W6D3 | BV711 |
| CD16 | BD/560474 | 3G8 | V450 |
| CD36 | BD/550956 | CB38 | APC |
| CD47 | BD/556046 | B6H12 | PE |
| CD62L | BD/563203 | DREG-56 | BV510 |
| CD11B | Beckman Coulter/ IM0530 | Bear1 | FITC |
| CD184 | BD/562389 | 12G5 | PE-CF594 |
| CD45 | BD/566041 | HI30 | APC-R700 |

**Supplementary Table 1**. List of antibodies used in FACS analysis.

**Supplementary Table 2.** Full clinical and pathological data on enrolled subjects.

| **ID** | **Age** | **Sex** | **Location** | **Smoking** | **pT** | **pN** |
| --- | --- | --- | --- | --- | --- | --- |
| 1 | 76 | F | T | No | T2 | N0 |
| 2 | 56 | F | T | Yes | T1 | N0 |
| 3 | 72 | M | T | Yes | T3 | N2c |
| 4 | 70 | F | T | No | T2 | N0 |
| 5 | 67 | M | T | No | T1 | N0 |
| 6 | 78 | F | T | No | T1 | N1 |
| 7 | 78 | M | T | Yes | T3 | N3b |
| 8 | 71 | M | T | Yes | T3 | N1 |
| 9 | 80 | M | G | No | T2 | N0 |
| 10 | 91 | F | G | No | T4a | N1 |
| 11 | 57 | M | T | No | T3 | N0 |
| 12 | 71 | M | T | No | T1 | N0 |
| 13 | 55 | M | B | Yes | T2 | N0 |
| 14 | 78 | F | G | No | T1 | N0 |
| 15 | 75 | F | T | Yes | T3 | N0 |
| 16 | 72 | M | T | Yes | T3 | N1 |
| 17 | 64 | M | T | Yes | T2 | N0 |
| 18 | 68 | M | T | Yes | T2 | N0 |
| 19 | 58 | F | G | Yes | T4a | N0 |
| 20 | 78 | F | T | Yes | T1 | N0 |
| 21 | 78 | M | T | No | T1 | N0 |
| 22 | 47 | M | T | no | T3 | N0 |
| 23 | 68 | F | G | N | T4a | N0 |
| 24 | 61 | M | T | Yes | T1 | N1 |
| 25 | 64 | F | G | Yes | T4a | N2b |
| 26 | 71 | M | G | Yes | T4a | N0 |
| 27 | 71 | F | B | Yes | T2 | N0 |
| 28 | 87 | M | G | Yes | T4a | N0 |
| 29 | 75 | F | T | No | T1 | N2b |
| 30 | 74 | F | F | No | T1 | N0 |
| 31 | 52 | M | T | Yes | T2 | N0 |
| 32 | 72 | F | B | No | T2 | N0 |
| 33 | 40 | M | T | No | T1 | N0 |
| 34 | 56 | F | T | Yes | T2 | N1 |
| 35 | 67 | M | F | Yes | T1 | N0 |
| 36 | 61 | F | T | Yes | T2 | N0 |
| 37 | 76 | M | F | Yes | T1 | N0 |

*Abbreviations: M – male, F – female, T – mobile tounge, G – gingiva, F – floor of the mounth, B – buccal mucosa*
